# Lymphatic Vascular Structures: A New Aspect in Proliferative Diabetic Retinopathy

**DOI:** 10.3390/ijms19124034

**Published:** 2018-12-13

**Authors:** Erika Gucciardo, Sirpa Loukovaara, Petri Salven, Kaisa Lehti

**Affiliations:** 1 Research Programs Unit, Genome-Scale Biology, Biomedicum Helsinki, University of Helsinki, FI-00014 Helsinki, Finland; 2 Unit of Vitreoretinal Surgery, Ophthalmology, University of Helsinki and Helsinki University Hospital, FI-00014 Helsinki, Finland; 3 Department of Pathology, University of Helsinki and Helsinki University Hospital, FI-00014 Helsinki, Finland; petri.salven@helsinki.fi; 4 Department of Microbiology, Tumor, and Cell Biology (MTC), Karolinska Institutet, SE-17165 Stockholm, Sweden; kaisa.lehti@ki.se

**Keywords:** angiogenesis, lymphatics, lymphangiogenesis, proliferative diabetic retinopathy, Lyve1, endothelial progenitor cell, ocular

## Abstract

Diabetic retinopathy (DR) is the most common diabetic microvascular complication and major cause of blindness in working-age adults. According to the level of microvascular degeneration and ischemic damage, DR is classified into non-proliferative DR (NPDR), and end-stage, proliferative DR (PDR). Despite advances in the disease etiology and pathogenesis, molecular understanding of end-stage PDR, characterized by ischemia- and inflammation-associated neovascularization and fibrosis, remains incomplete due to the limited availability of ideal clinical samples and experimental research models. Since a great portion of patients do not benefit from current treatments, improved therapies are essential. DR is known to be a complex and multifactorial disease featuring the interplay of microvascular, neurodegenerative, metabolic, genetic/epigenetic, immunological, and inflammation-related factors. Particularly, deeper knowledge on the mechanisms and pathophysiology of most advanced PDR is critical. Lymphatic-like vessel formation coupled with abnormal endothelial differentiation and progenitor cell involvement in the neovascularization associated with PDR are novel recent findings which hold potential for improved DR treatment. Understanding the underlying mechanisms of PDR pathogenesis is therefore crucial. To this goal, multidisciplinary approaches and new ex vivo models have been developed for a more comprehensive molecular, cellular and tissue-level understanding of the disease. This is the first step to gain the needed information on how PDR can be better evaluated, stratified, and treated.

## 1. Introduction

The global prevalence of diabetes has nearly doubled in the past three decades, with 425 million individuals affected in 2017 [1]. Concomitantly, the prevalence of the most common microvascular complication of diabetes, diabetic retinopathy (DR), has also increased. DR is a multifactorial disease involving the complex interplay of microvascular, neurodegenerative, metabolic, genetic/epigenetic, immunological, and inflammation-related factors. According to the level of microvascular degeneration and ischemic damage, DR is classified into non-proliferative DR (NPDR) and proliferative DR (PDR). Diabetic macular edema (DME), the accumulation of extracellular fluid within the retinal layers around the macular region, often accompanies all stages of DR. The end-stage disease, PDR, is characterized by ischemia- and inflammation-induced neovascularization, coupled with fibrotic responses at the vitreoretinal interface, which, in untreated conditions leads to blindness due to vitreous hemorrhage (VH), retinal fibrosis, tractional retinal detachment (TRD), and neovascular glaucoma [2,3,4]. PDR pathogenesis also involves injury of neurons and glial cells, dysfunction of endothelial progenitor cells (EPCs) and accumulation of inflammatory cells [2,5,6].

## 2. The Vitreal Microenvironment in Proliferative Diabetic Retinopathy

The vitreous fluid, rich of extracellular matrix (ECM), soluble proteins, and macromolecules, reproduces the soluble microenvironment of growth factors, cytokines, and metabolites within the posterior eye chamber and, as such, is useful to indirectly investigate the ongoing pathological processes taking place in the diabetic retina [7]. Increased intravitreal factors in PDR eyes may be released from the different cell types, including ischemic and/or hypoxic retinal vascular ECs, retinal pigment epithelial (RPE) cells, pericytes, fibroblasts, inflammatory infiltrates, reactive glial cells, and injured or dying neurons, or diffuse from plasma through the leaky vasculature [8,9,10,11,12].

### 2.1. Angiogenesis-Promoting Factors

In the seek for the factors responsible for PDR pathological neovascularization, research efforts have focused on quantifying and comparing the levels of selected angiogenic factors between DR stages, as well as other retinal diseases [13]. Proangiogenic factors like vascular endothelial growth factor-A (VEGFA), angiopoietin-2 (Ang2), platelet-derived growth factor (PDGF), basic fibroblast growth factor (bFGF), osteopontin (OPN), erythropoietin (EPO), stromal cell derived factor-1 (SDF1), cysteine-rich 61 (CYR61) have been found at elevated levels in PDR vitreous [14]. Interleukin-37 (IL-37), also recently suggested as a novel proangiogenic cytokine, increases in PDR vitreous [15]. Angiogenesis effectors such as soluble matrix metalloproteinase-2 (MMP2) and MMP9 are also present at higher levels in PDR than in NPDR [16,17]. Consistent with the angiogenic nature of this diabetic complication, anti-angiogenic factors, such as pigment epithelium-derived factor (PEDF), are reduced in PDR [18].

Among all angiogenesis regulators, VEGFA has been most extensively studied and provides the basis for current anti-angiogenic therapy [19]. VEGFA plays a crucial role in PDR pathogenesis by promoting vascular leakage and neovascularization. VEGFA binds to its main receptor vascular endothelial growth factor receptor-2 (VEGFR2) expressed on ECs and induces dissociation of tight junctions, proliferation and sprouting angiogenesis. Although current targeted therapies can inhibit these VEGFA functions, the anti-VEGFA therapy is not effective for all patients. Notably, not all PDR patients have detectable and/or high vitreal VEGFA, suggesting that, despite the function of VEGFA in DR progression is indisputable, VEGFA-independent pathways also contribute to PDR development [7,20]. Identifying these pathways and their promoting factors will guide the development of new therapies against DR progression [21]. Mass spectrometry-based characterization of the vitreous proteome has recently brought into light processes, such as inflammation, coagulation, and complement activation, among others, thus widening the understanding of DR pathogenesis beyond the mere pathological angiogenesis [7,22].

### 2.2. Inflammation-Promoting Factors

Oxidized lipoproteins, advanced glycation end-products and free radicals that form as a result of chronic hyperglycemia, are considered major causes of tissue stress and serve as local triggers for inflammation, affecting virtually every cell type in the retina, including the vasculature [23,24]. Chronic exposure to these stress factors, together with distressed and/or deregulated homeostasis, results in chronic inflammation which contributes to DR progression [25,26,27,28]. Hyperglycemia-induced oxidative stress induces NF-κB activation, followed by production of inflammatory cytokines, chemokines, and cell adhesion molecules, as well as recruitment of inflammatory cells to the retinal tissue [29,30]. Several inflammatory factors have been found at elevated levels in PDR vitreous fluid, including IL-1β, IL-6, IL-8, IL-10, IL-18, tumor necrosis factor-α (TNFα), monocyte chemoattractant protein-1 (MCP-1), macrophage migration inhibitory factor (MIF), macrophage-colony stimulating factor (M-CSF), high mobility group box-1 (HMGB1), and nucleotide-binding oligomerization domain, leucine rich repeat and pyrin domain containing-3 (NLRP3) inflammasome [31,32,33,34]. The activated angiogenic endothelium represents a major source of these factors. Inflammatory cells recruited to the diseased retina in turn produce angiogenic cytokines and effectors, activating a wound healing response coupled with non-productive angiogenesis. This abnormal response, rather than fulfilling oxygenation and metabolic demands, leads to hemorrhage, hypoxia, fibrosis, further tissue damage, and overt neovascularization. The mechanistic link between angiogenesis and inflammation is, thus, complex as these processes are both individually activated and reciprocally intertwined, while sharing common mediators and pathways [35].

### 2.3. Factors Involved in Lymphatic Vascular Formation

The development and growth of new lymphatic vessels is highly regulated via a balance between various activators and inhibitors [36,37]. Factors that could drive angiogenesis in PDR eyes by virtue of their increased vitreal levels compared to NPDR, such as VEGFA, Ang2, bFGF, NLRP3, MMP2, MMP9, TNFα, and IL-1β, are also capable of driving lymphangiogenesis in vivo [16,38]. The mechanisms for the induction of lymphatic neovascular formation can be direct by e.g., effects on existing lymphatic vasculature or EPCs, or indirect through, e.g., the induction of VEGFC expression or recruitment of VEGFC- and VEGFD-producing macrophages [39]. VEGFC and VEGFD are major drivers of both developmental and adult physiological and pathological lymphangiogenesis. They exert their function through binding to VEGFR3 and induce lymphatic endothelial cell (LEC) proliferation, migration, and survival [40]. Mature forms of VEGFC and VEGFD also bind and activate VEGFR2, a mechanism not yet deeply elucidated [41,42].

The involvement of VEGFC and VEGFD lymphangiogenic growth factors in eye diseases and diabetic complications, in general, has not been investigated, until recently. In diabetic kidney disease, VEGFC induced lymphangiogenesis in association with fibrosis through transforming growth factor-β (TGFβ) and connective tissue growth factor (CTGF) mediated upregulation of VEGFC expression [43]. While this effect could be detrimental for kidney function, leukocyte trafficking and ECM deposition during local lymphangiogenesis induced by VEGFC improves healing of diabetic wounds [44]. Interestingly, VEGFC and VEGFD have also been detected in the vitreous fluids of PDR patients [13,45]. Thus, taking these factors altogether, the complex pathological microenvironment of PDR eyes represents a plausible niche for lymphatic-like neovascular formation.

**Figure 1:**
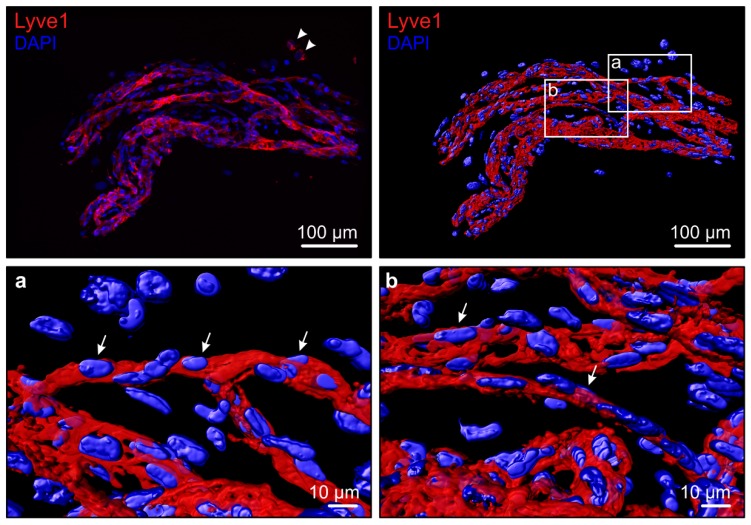
3D volume reconstruction of a fresh PDR fibrovascular tissue shows lymphatic endothelium. The PDR fibrovascular tissue was stained by whole-mount immunofluorescence for Lyve1 (R&D Systems, Oxon, UK) (red). Hoechst-33342 (Thermo Fisher Scientific, Cheshire, UK) counterstain (blue) visualizes nuclei. Arrows indicate the Lyve1+ endothelial cell lining in the PDR neovasculature. Isolated Lyve1^+^ positive extravascular cells, possibly macrophages, are also present (arrowhead). The image was taken using Zeiss AxioImager.Z1 upright epifluorescence microscope with Apotome combined with a computer-controlled Hamamatsu Orca R2 1.3 megapixel monochrome CCD camera and ZEN software, using a 20× Plan Apochromat, 0.8 NA objective (Zeiss, Jena, Germany). The captured Z-stack was imported into Imaris software (Imaris, version 9.1.0, Bitplane, Zurich, Switzerland) and processed by volume and surface reconstruction of the Lyve1^+^ endothelium and of the nuclei.

The lymphatic vascular formation in the ischemia- and inflammation-induced neovascularization of PDR is a recent novel finding (Figure 1 and Supplementary Video) [2,45]. In a recent report, VEGFC levels did not correlate with PDR progression [13]. Yet, we found that LEC 3D sprouting is induced by vitreous fluid in a VEGFC level-associated fashion [45]. The maintenance of lymphatic capillaries is dependent on constant VEGFC signaling [46]. We found that in the patient-matched surgically-excised fibrovascular PDR tissues, vitreous-induced LEC sprouting is also associated with lymphatic marker expression [45]. Therefore, the complex ischemic and inflammatory PDR microenvironment, rather than a single factor, is likely to drive lymphatic-like vessel formation. VEGFC is commonly produced by pericytes, smooth muscle cells, cells in the pituitary and pineal glands as well as by macrophages, and its production is induced by pro-inflammatory cytokines [47,48]. While some of the VEGFC-producing cells are present in the retina, the cellular source of vitreal VEGFC in PDR is currently unknown.

## 3. Physiological Lymphatic Vasculature in Ocular Structures

Research on the existence of a lymphatic system/drainage in brain and eye in human has been limited due to difficulties to solidly identify lymphatic vessels as well as to access healthy and/or developmental tissue material. Recently, the establishment of a set of specific LEC markers, such as VEGFR3, lymphatic vessel endothelial hyaluronan receptor-1 (Lyve1), prospero homeobox protein-1 (Prox1) and podoplanin (PDPN) has enabled great progress in the understanding of lymphatic vascular biology [49,50]. Arisen controversies concerning the existence of “classical” lymphatics in the eye led in 2014 to the establishment of a consensus for the immunohistochemical detection of ocular lymphatic vessels [50]. However, contradictory findings have also been reported thereafter. Positive and negative findings on the existence of lymphatic vessels in ocular structures in physiological conditions are summarized in Table 1.

In adult physiological conditions, lymphatic vessels have been consistently found in the conjunctiva, iris, limbus, ciliary body, and orbital meninges [46,51,52]. By virtue of the absence of the above-mentioned LEC markers in lumen-lined structures, the retina, sclera, and cornea have instead been considered to lack lymphatic vessels in physiological conditions [50,53]. Structures like the sclera and cornea that lack typical lymphatic vessels have instead been found to be endowed with Lyve1^+^ macrophages [53,54]. In the retina such resident Lyve1^+^ macrophages are absent. Instead microglial cells with different sets of markers are a form of specialized retinal tissue resident cells performing similar scavenging and homeostatic functions. Within choroid, Lyve1^+^ macrophages have been consistently found, while the presence of lymphatic vessels remains highly debated [55,56,57,58]. With clearly distinctive features, the Schlemm’s canal, an endothelium-lined vessel encircling the cornea, has also been recently recognized as a lymphatic-like vessel by virtue of its expression of Prox1 [46].

Using histological criteria and single-label immunohistochemistry, lymphatic vessels have also been reported in the optic nerve (ON) and ON sheaths in adults [59,60]. Latest findings, however, reported a lack of lymphatic vessels, while documenting the presence of Lyve1^+^ macrophages, which is in accordance with findings in human fetal eyes [61,62]. Therefore, the question on whether the ON and ON sheaths contain lymphatic vessels has not been yet clearly answered. Lymphatic drainage through the ON region was reported in rabbits using ink injection experiments [63]. In adult mouse a dural lymphatic vasculature running along the dural sinuses forms a system for circulation and drainage of cerebrospinal fluid (CSF), and lymphatic vessels are also observed around the ON postnatally [48]. However, the limitation with using animal models is that, in as much as they are instrumental for answering the developmental/physiological pressing questions, they are anatomically different from human, for what pertains the eye. Therefore, findings in these model systems cannot be directly translated to human. For example, rabbits have no lamina cribrosa whereas mice lack the macula, a region that in humans becomes edematous during DR progression. A system for lymphatic drainage has instead been described for the anterior eye segment, whereby aqueous humor is drained through trabecular meshwork and Schlemm’s canal, through the uveoscleral pathway, or through ciliary body lymphatics [52].

## 4. Lymphatic Vascular Formation in Ocular Diseases

Lymphatic neovascularization is a key event in acute and chronic inflammation, as well as in tumor progression. Pathological lymphatic neovascularization occurs in several diseases involving the anterior eye segment, e.g., in cornea upon corneal transplant rejection [64], in uvea/scleral border and conjunctiva upon uveal melanoma progression [69,77], and in cornea and sclera upon wound healing and ocular trauma [64,73]. For reviews, see [36]. While investigating the presence of lymphatic vessels in the human optic nerve, Gausas et al., utilized ten eyes enucleated due to end-stage glaucoma or retinal diseases and documented PDPN-positive vascular structures within the dura mater in two out of 10 specimens [70]. The authors reasoned that undetected lymphatics in majority of the specimens could be due to disease-associated alterations. The appearance of lymphatic vessels in this pathological condition is, however, likely relevant to glaucoma pathophysiology.

In human pathological conditions of the posterior eye segment, we first reported the lymphatic vessel involvement based on immunohistochemical and electron microscopic observations of lymphatic-like vascular structures in patient-derived fibrovascular tissues surgically-excised from PDR eyes [2]. The PDR neovasculature contained, together with blood vessels, erythrocytes-free vessels expressing the lymphatic markers Lyve1, Prox1, and VEGFR3. Later 3D characterization and RNA sequencing confirmed the expression of these markers [45]. Consistent with the abnormal nature of the PDR neovascular structures, the lymphatic marker PDPN was not co-expressed in the vessels [2].

Lymphatic-like vessels expressing Lyve1 and Prox1 were also found in the neovascularization associated with a case of retinal vein occlusion (RVO) [78]. Neovascular (hemi) RVO is a vascular occlusive condition characterized by shorter, more acute, ischemia compared to PDR. Yet, the metabolic imbalance in this RVO patient case confirms the open questions regarding the factors involved in lymphatic-like neovascular formation, as well as the link between inflammation and ischemia and its outcomes in complex and multifactorial retinal diseases, including RVO and PDR [13]. Together with ischemia, PDR involves edema formation and chronic inflammation, which are both triggers for lymphangiogenesis. As the demand for fluid drainage and immune/inflammatory cell trafficking increases, it is plausible that a lymphatic-like system can emerge during pathological conditions of the posterior eye segment [79,80].

## 5. Mechanisms of Pathological Lymphatic Vascular Formation

Investigating the cellular and molecular mechanisms driving lymphatic-like neovascular formation in ischemia- and inflammation-driven diseases of the retina is required in order to better understand PDR pathophysiology and possibly reconsider the therapeutic targeting of this devastating eye disease. In general, lymphatic neovascularization can occur by lymphangiogenesis from pre-existing vessels or de novo through a process more similar to lymph-vasculogenesis.

### 5.1. Lymphatic Vascular Formation from Pre-Existing Vessels

Lymphatic vessels can arise through invasion of new areas by pre-existing lymphatic vessels, in response to lymphangiogenic signals. This is the case for the anterior eye segment where lymphatics exist in physiological conditions. In cornea, pathological lymphangiogenesis occurs from pre-existing limbal lymphatics during keratitis and trauma [64]. In uveal melanoma with extraocular extension, lymphatic vessels have been proposed to invade from pre-existing conjunctival lymphatics [77]. In a similar manner, pre-existing limbal and/or conjunctival lymphatic vessels can outgrow upon ocular trauma [73]. In the posterior eye segment, conventional lymphatics from which pathological lymphangiogenesis could occur have not been consistently found.

**Figure 2:**
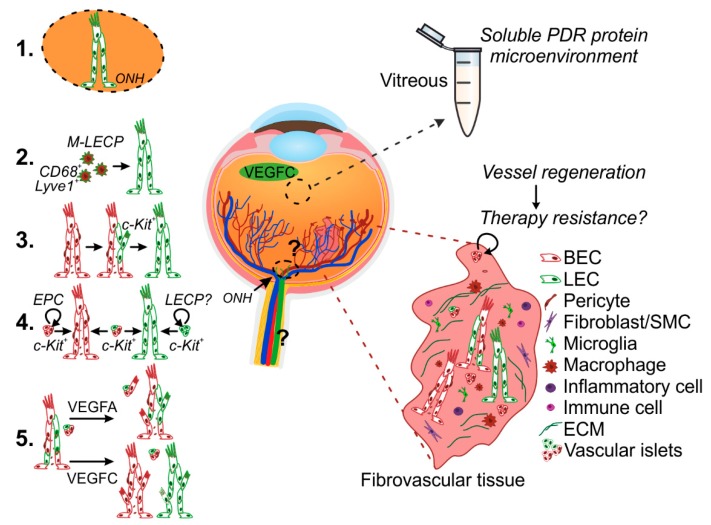
Potential mechanisms of pathological lymphatic vascular formation in PDR. Lymphatic vessel formation was recently discovered in the ischemia- and inflammation-induced neovascularization occurring in PDR [2,45]. The vitreous fluid collected during vitrectomy reproduces the soluble microenvironment of growth factors, cytokines, and metabolites within the posterior eye chamber and is utilized to indirectly investigate the ongoing pathological processes in the diabetic retina. The patient-derived fibrovascular tissue, excised during vitreoretinal surgery, provides invaluable material for understanding PDR pathophysiology in the complexity of the live human tissue microenvironment. The complex ischemic and inflammatory PDR microenvironment, including VEGFC detected in vitreous, can drive PDR lymphatic vascular formation. Lymphatic vascular formation is essentially achieved through pathological lymphangiogenesis from pre-existing lymphatic vessels and/or de novo, lymph-vasculogenesis. The question on whether the human optic nerve and retinal regions include yet uncovered lymphatic vasculature remains to be investigated (1). Macrophages which have the ability to transform into LECs (M-LECPs) can contribute to de novo lymphatic vascular formation (2). Alternatively, lymphatic neovascularization could occur through trans-differentiation from blood endothelial cells (BECs) (3) or through LEC fate induction in resident or incoming EPCs (4). Sphere-like vascular cell structures interspersed within the PDR fibrovascular tissues could be the vessel primordia for neo (lymph) vascularization. This could also provide a mechanism for vessel regeneration and thus be responsible of re-vascularization after treatment (therapy resistance). The abnormal PDR neovasculature displays heterogeneous population of abnormally differentiated vessels [45]. Ex vivo culture led to heterogeneous vessels and islet sprouting in presence of VEGFA, while VEGFC promoted segregation of the heterogeneous vasculature into separate vessels (5) [45]. More in-depth studies are needed in order to understand the signals and mechanisms involved in the pathological neovascularization observed in PDR. ONH, optic nerve head; BEC, blood endothelial cell; LEC, lymphatic endothelial cell; SMC, smooth muscle cell; LEC-P, LEC-precursor; M-LECP, macrophage-derived LEC-P.

Retinal neovascularization in PDR occurs at the optic nerve region (NVD) and in the peripheral retina (NVE). The optic nerve region contains a dense vascular plexus, formed by branches of the ophthalmic artery, posterior ciliary arteries, and central retinal artery and vein, which run through the dura and pia mater and emerge from the optic nerve head to vascularize the entire retina. Whether the ON is in the neighborhood of yet uncovered lymphatic vasculature, while being richly vascularized by arteries and veins, is a pressing question (Figure 2). In mice, a lymphatic vasculature runs adjacent to the brain dural blood vasculature and exits the skull along arteries, veins, and most cranial nerves, including the ON [81]. Anatomically, the dura mater of the ON is continuous with the dural meningeal sheath that covers the entire brain parenchyma. Whether an analogous anatomical arrangement of blood vessels and lymphatic-like vessels exists along the ON in humans and whether, together with the lymphangiogenic vitreal microenvironment, this becomes relevant during DR progression remains to be investigated.

### 5.2. De Novo Lymphatic Vascular Formation–Macrophages

Macrophages have been described to contribute to de novo lymphatic vascular formation through their ability to transform into LECs and integrate as tubular structures [82,83,84]. Resident macrophages exist in many ocular locations, including the cornea, sclera, iris, choroid, and ON sheaths [57,85,86]. They support homeostatic functions and carry out inflammatory responses to pathogenic stimuli [85]. For example, in the cornea they facilitate injury repair by de-differentiating into keratocytes and contributing to collagen synthesis [87].

In addition to these functions, resident and recruited macrophages in mouse give rise de novo to Lyve1^+^ tubular structures during corneal inflammation-induced lymphangiogenesis [82]. Accordingly, macrophage depletion leads to reduced lymphatic vessels formation in a corneal suture model of inflammation [88]. In melanoma of the ciliary body with extraocular extension compromising the scleral border, Prox1^+^ macrophages can invade new areas from the sclera and conjunctiva, and give rise to lymphatic vascular structures [69,77,82].

Macrophage-derived LEC precursors (M-LECP) co-express myeloid/macrophage markers, such as CD68 and CD11b, and the established LEC markers Prox1, Lyve1, VEGFR3, and podoplanin. Double-positive (macrophage and LEC markers) cells of this kind reside in the sclera, iris, choroid, and ON, suggesting lymph-vasculogenesis from resident macrophages as a possible/alternative mechanism for the lymphatic vascular formation observed in ocular neovascularization. The retina is populated by a specialized type of resident macrophages, called microglia, and by a smaller population of perivascular macrophages [89]. Extra/perivascular cells expressing Lyve1 and VEGFR3 occurred in the PDR fibrovascular tissues. CD68^+^ macrophages also existed, especially in areas of highly unorganized endothelium [2,45]. The original source and direct contribution of these cells to de novo lymphatic vascular formation in PDR remains to be thoroughly dissected (Figure 2). Therefore, whether these PDR tissue macrophages represent LEC precursors or their contribution to lymphatic-like vascular formation, if any, can rather be limited to lymphangiogenesis induction via VEGFC and VEGFD production needs to be investigated. In a mouse model of suture-induced inflammatory corneal neovascularization, recruited macrophages induce lymphangiogenesis via production of VEGFC and VEGFD [39]. Interestingly, VEGFC has also been reported to induce ON oligodendrocyte precursor cell (OPC) proliferation, opening questions on the multiple effects that the VEGFC found in PDR vitreous can have on the identity of different resident and/or recruited cell types within the PDR tissue [90].

Upon vitreous traction, the subretinal space, outer to the RPE and inner to the Bruch’s membrane and choroid, becomes exposed to the vitreous cavity, thus offering a route for microglia/macrophage retinal trafficking [91]. Although Lyve1^+^ macrophages infiltrate the mouse retina during choroidal neovascularization, typical lymphatic structures have not been observed [92]. The human choroid contains Lyve1^+^/CD68^+^ positive macrophages, whose possible contribution to PDR neovascular formation also remains to be investigated [57].

### 5.3. De Novo Lymphatic Vessel Formation—Endothelial Progenitor Cells (EPCs)

Alternatively, lymphatic vessels can arise de novo from EPCs through a process similar with developmental lymph-vasculogenesis (Figure 2). During development, the majority of first LECs are generated through trans-differentiation of venous blood and ECs upon upregulation of Prox1. Recent lineage tracing experiments also revealed that a substantial part of the lymphatic vasculature in dermis is formed from non-venous progenitors of non-hematopoietic origin, through clustering and coalescence into vessel networks [93]. In addition, a population of c-Kit^+^ cells of hemogenic endothelial origin has been found to give rise to mesenteric lymphatic vessels [94]. These findings, while excluding the direct contribution of macrophages for such developmental lymph-vasculogenesis, open questions on the identity/origin and potential functional significance of lymphatic precursor cells in adult physiological and pathological lymphatic vascular formation.

While being well-defined for blood endothelium, the origin of LECPs and their contribution to pathological lymphatic neovascularization are not completely understood. The adult blood vessel wall endothelium contains a subpopulation of c-Kit^+^ resident vascular endothelial stem cells (VESC) that act as EPCs and contribute to adult neovascularization [95]. In this scenario, LECs could result from LEC fate induction of the retinal (neo)vessel wall-resident activated progenitors or through trans-differentiation from abnormal/angiogenic BECs (Figure 2). Vessel wall-resident EPCs analogous to VESCs have not been found in lymphatic capillaries, whereas studies have shown that the bone marrow contains cells with such potential [96].

The involvement of resident or incoming c-Kit^+^ EPCs in the pathophysiology of PDR has been described by us and other researchers on thin histological sections [2,97]. How these cells contribute to PDR neovascularization is not completely understood. However, by 3D characterization of the PDR neovasculature, we identified unusual, CD31^+^ and ERG^+^ sphere-like vascular structures reminiscent of vessel primordia. While lymphatic marker expression in these relatively rare structures remains to be investigated, their increase along with vessel preservation, induced by VEGFC, supports their potential for lymphatic neovascularization [45]. These structures could also provide a mechanism for vessel regeneration and, thus, be responsible of re-vascularization after treatment, with implications on therapy response.

Altogether, these mechanisms are in accordance with the heterogeneous nature of the abnormal PDR neovasculature, including the discontinuous expression of canonical lymphatic markers and the distinct responses to angiogenic factors, such as VEGFA and VEGFC (Figure 2) [45]. Deeper understanding on LECPs and on the possible involvement of BEC progenitors in PDR lymphatic neovascularization will open new areas of investigation into organ-based lymphatics and LEC fate during adult pathological settings. Moreover, future insights on the ability of the distinct PDR cells, recruited to the pathological tissue locally or via circulation, to become transcriptionally reprogrammed, and to induce lymphangionenesis or lymph-vasculogenesis, can help us to understand whether the lymphatic-like vessels develop coincidentally/constantly with abnormal blood vessels or only later upon PDR progression. In a model of corneal neovascularization, inflammation-associated angiogenesis and lymphangiogenesis occur concomitantly via the action of VEGFA and can be inhibited by Bevacizumab, thus warranting more in-depth studies into the molecular signals and mechanisms involved in posterior eye segment (lymph)neovascularization [39,98].

## 6. Experimental Models of Diabetic Retinopathy

The animal models of diabetes, oxygen-induced retinopathy (OIR) and pericyte-depletion developed to date have been instrumental in understanding the pathophysiology of diabetic retinopathy [99,100,101,102]. The OIR and pericyte-depletion models reproduce features of preclinical human disease, including vascular regression, hypoxia-induced angiogenesis, leukostasis and neuronal degeneration but lack features of the advanced proliferative disease. Diabetic mouse models also develop many features of early stage DR in a hyperglycemic background but, like the other models, do not develop prominent PDR.

Hyperglycemia, hypoxia, and chronic inflammation are the three major pathogenic processes that characterize PDR. Current models are hyperglycemia- or hypoxia-only models and lack the contribution of chronic inflammation, central to PDR pathogenesis. A model combining at least two of these mechanisms was developed by intravitreal injection of pro-inflammatory cytokines in non-obese diabetic (NOD) mice [103]. NOD mice spontaneously develop type 1 diabetes and features of human NPDR. Intravitreal injection of pro-inflammatory cytokines that are commonly found at increased levels in human vitreous, lead to additional, PDR, features. Interestingly, the injected cytokines induced reactive changes in astrocytes and macrophages/microglia that localized mainly at the ON head. Such a feature would make this model useful for investigating more in detail hyperglycemia- and inflammation-induced changes taking place in the ON region, in relation to lymphatic neovascular formation, yet this model does not develop the proliferative neovascular changes manifested in human PDR [103]. This may be due to the acute inflammation induced in this model, differently from the chronic inflammation occurring in human NPDR and PDR, where pathological changes in the retina develop over decades.

Models of pericyte depletion/damage have also been used for investigating PDR development based on the idea that pericyte loss occurs early in PDR and is a common trigger for an angiogenic response. While being instrumental for understanding the molecular mechanisms of pericyte loss occurring in PDR, as well as in other vascular degenerative diseases, this model explores just one of the cellular/microenvironmental compartments in the retina, whereas DR pathological changes also involve the neural and ECM components of the retina, in addition to the vasculature [4,29,104]. Moreover it is increasingly clear that more than the loss of pericyte, the EC-pericyte signalling alterations are rather relevant for DR progression [105].

Other animal models also serve as powerful tools for studying angiogenesis and lymphangiogenesis [106]. In the Prox1-GFP/Flt1-DsRed (PGFD) model, researchers used reporter systems to label blood and lymphatic vessels for direct visualization. For the first time researchers analyzed the formation, branching, and regression of both blood and lymphatic vessel types in a live mouse cornea throughout an experimental time course. While specific inherent labeling of blood and lymphatic vessels could aid the characterization of the pathological retinal neovasculature, current animal models of diabetes need further improvement in recapitulating PDR proliferative changes before they can be combined with this in vivo labelling approach. Moreover, the location and accessibility of the retinal tissue poses challenges for in vivo studies, differently from the readily accessible cornea or superficial lymphatic vascular networks.

Indocyanine green fluorescence lymphography has been used to comprehensively map the superficial lymphatic system in the rat [107]. Photoacoustic imaging can instead be used for non-invasive visualization of deep lymphatic vessels [108]. Additionally, most recent high-resolution brain magnetic resonance imaging units might achieve visualization of blood and lymphatic vasculature in ocular structures, including ON and retina.

Injections of fluorescent tracers and in vivo microscopy have been used in the studies of meningeal lymphatic system [81,109,110]. Unfortunately, the in vivo visualization of the human lymphatic system is limited by the mode of delivery of tracer agents [111]. Developing less invasive methods to image human eye lymphatics will also constitute a major advance in eye research. Currently, non-invasive means of assessing lymphatic function in severely diseased PDR eyes are non-existent.

While mouse models are useful tools to, e.g., study cancer disease etiology and pathogenesis, the murine eye is fundamentally different from the human eye, in that it lacks the macula [112]. Therefore, no mouse model can, e.g., fully recapitulate macular edema, a feature that is central to PDR pathophysiology, especially in light of most recent findings that call lymphatic drainage into the PDR scene [113]. Furthermore, what is known about the expression of LEC markers podoplanin, Lyve1, Prox-1, VEGFR3 in rodent lymphatic vessels and human LECs may not apply in human lymphatic disease settings in different organs [111]. Many concepts can be borrowed from the lessons learned in other organs, although there are also organ-specific mechanisms [114]. For example, lymphatic marker Lyve1 is a specific receptor for hyaluronic acid (HA), an element of vitreous gel which also regulates cell migration in the course of wound healing and inflammation.

Invaluable material for advancing the understanding of PDR pathophysiology, as well as for testing treatment responses in the complexity of human tissue microenvironment is provided by patient-derived neo(fibro)vascular PDR tissue. We recently developed a novel ex vivo method for patient-derived neo(fibro)vascular tissue culture, as a model for human PDR pathophysiology [45]. Using this model, we discovered prominent 3D in vivo patterns of Lyve1, Prox1, and VEGFR3 lymphatic marker-positive vascular structures. These structures contained abnormally differentiated capillaries with heterogeneous mixtures of cells with abnormal BEC and LEC identity, but also structures resembling more mature lymphatic vessels. This highlights the need for investigation of possible functionality and specific contribution of these LEC-invested vessels to PDR pathophysiology [45]. Generally, this ex vivo model can be used for the simultaneous assessment of molecular mechanisms and treatment responses in the context of integrated communication among multiple cell types upon processes related to (1) inflammation and (2) pathological angiogenesis and lymphatic-like endothelial differentiation, coupled to (3) metabolic imbalance and (4) fibrosis.

## 7. Multimodal Imaging and Improved Surgical Technology

During the last few decades, evaluation of clinical characteristics in NPDR and PDR eyes has been performed together with morphological investigation [3,115]. Innovations in computing technology related to structural retinal analysis such as spectral domain optical coherence tomography (SD-OCT) and OCT-angiography (OCTA) have helped scientists and clinicians to assess, evaluate and manage the pathological changes occurring in human retina and choroid [116]. Use of multimodal OCTA has been rapidly expanding in clinical practice and in research of posterior pole retinal diseases, including PDR [117]. OCTA enables clear visualization of the retinal vasculature, including the superficial and deep retinal plexuses, as well as of the PDR fibrovascular formation above the optic disc [117,118]. Recent OCTA findings demonstrated that the locations of the NVDs were outside the physiological cup, supporting the speculation that NVDs may perfuse through the choroidal component of the microcirculatory system of the optic disc [117]. Enhanced depth imaging-optical coherence tomography (EDI-OCT) and swept-source OCT can help shed more light into the choroidal and macular changes in diabetic eyes [119].

Additionally, great technological advances in the vitrectomy technique have been achieved, including new microincisional vitrectomy technology, 3D viewing heads-up vitreoretinal surgery, wide-angle microscope viewing systems, intraoperative OCT imaging, pharmacologic chromovitrectomy, and use of anti-VEGF therapeutic agents [120,121,122]. All these technological advances will allow more accurate analysis of PDR membranes peri-operatively and improve facilities to surgically collect these invaluable PDR membranes for scientific use, while improving PDR patient outcomes.

## 8. Clinical Interventions and Potential Novel Drug Targets

Treatments for PDR have greatly improved over the past decade [123,124]. PDR treatment includes vitrectomy, panretinal photocoagulation (PRP), and intravitreal anti-vascular endothelial growth factor (VEGF). Vitrectomy is performed to clear vitreous hemorrhage, relieve retinal traction and for fibrovascular tissue removal. PRP, the thermal destruction of the ischemic retinal tissue, is the standard-of-care for advanced NPDR and PDR despite its inherent substantial side effects on visual field, night vision, retinal fibrosis, epiretinal membrane formation, and macular edema [125]. Intravitreal anti-VEGF, administered when PRP cannot be performed due to, e.g., VH or cataract, as well as preoperatively (usually 3–7 days before vitrectomy) as an adjunct treatment, reduces intravitreal bleeding through rapid involution of the active neovascularization, and decreases vascular perfusion and permeability, thus facilitating the surgical excision of the fibrovascular PDR tissue [126,127]. In light of recent clinical trials, intravitreal anti-VEGF agents have also been recognized as alternative treatment options to PRP [128,129]. Although anti-VEGF offers lower incidence of vision-impairing macular edema and less visual field loss compared to PRP, a substantial proportion of patients will still require vitreoretinal surgery due to further development of complications such as VH, macular edema or fibrotic responses causing TRD [123,129,130]. The involution of the neovascular formation achieved by anti-VEGFA treatment is indeed often transient and the absence of other complications remains an open critical question.

Altogether, current treatment options target only advanced NPDR and PDR stages, when retinal damage has already ensued. Early detection of diabetes, early and regular screening of retinopathy, blood pressure, lipidemic, and glycemic control remain the only available options aimed at preventing or arresting the progression of DR [131,132,133].

Numerous new therapies for retinal neovascular complications are under active investigation. In Table 2, we list pharmacologic intervention-only studies that we found in clinicaltrials.gov in November 2018 using “neovascularization, retina” as search term, after excluding studies on conventional agents used in clinical practice, as well as studies that were terminated, withdrawn, suspended or with unknown status. This search retrieved 19 studies. Currently, there are also other potential DME/DR drugs under development that might change the clinical practice for ocular neovascular diabetic complications. Essentially, new drugs related to tyrosine kinase signaling, integrin signaling, Tie 2 pathway (anti-Ang2), plasma kallikrein system, Sema3A system and MMP-2 and MMP9 blockade are under intensive investigation [134,135,136,137,138]. Anti- oxidants, anti- inflammatory drugs, angiotensin-converting enzyme (ACE) inhibitors, protein kinase C (PKC) inhibitors, as well as stem cell therapies for repairing the injured retinal microvessels are also currently being considered [3,139,140,141,142]. In the near future, the question whether administration of VEGFC could be therapeutic and promote healing in ischemic and fibrotic PDR eyes, not only in ischemic heart, should be solved [114,143,144]. Nanoparticles also hold great potential to shape the future of ocular anti-angiogenic therapy in eyes with PDR [145,146].

Discovery of novel, more effective and tailored treatments will ultimately rely heavily on findings from clinically relevant functional models used with or without the existing mouse models, in vitro cell culture studies, and clinical and biochemical data obtained with proteomics analyses [7,45]. Since ocular neovascularization, including PDR, often includes both blood and lymphatic networks, these two biological systems should be studied together [147].

## 9. Conclusions and Future Directions

Despite significant advances in the knowledge on the PDR vitreous composition, the factors behind PDR pathogenesis, including inflammation, wound healing, and pathological angiogenesis, remain incompletely understood [6,7]. To understand the importance of blood and lymphatic vascular mechanisms as well as the contribution of other cell types, including inflammatory and immune cells in PDR pathophysiology, thorough characterization of neovessel and fibrovascular tissue properties of PDR tissue will be essential together with systematic mechanistic and functional studies [45,148,149]. Improved diabetic mouse models recapitulating in concert inflammation, hyperglycemia and hypoxia, can be instrumental. Optimally, lineage tracing approaches in mouse models will be highly beneficial to understand the fate of the different retinal resident and incoming cell types and their contribution to the lymphatic-like neovascular structures arising in PDR.

Understanding both angiogenic and lymphangiogenic mechanisms responsible for initiation, progression and resolution of retinal fibrosis associated with DR/PDR is also crucial to design novel treatments. Perhaps, stimulation of lymphangiogenesis in PDR will be beneficial and result in enhanced resolution of inflammation. However, little is currently known of the role of lymphatics in the pathogenesis of PDR or how modulation of lymphangiogenesis can affect DR/PDR progression [2,45]. Remodeling of vasculature could also differ due to different environmental or epigenetic factors (such as smoking, use of medication, concomitant systemic disease). Therefore, the roles of pro-lymphangiogenic and anti-lymphangiogenic factors in PDR inflammation deserve to be characterized in the future.

The lymphatic endothelial involvement and Prox1 expression in the PDR neovessels will be of interest also regarding the presence of lymphatics along the ON [45]. In general, answering questions on the presence of lymphatics in the human ON and mechanisms of drainage at posterior eye segment will require the use of human post-mortem material with strict inclusion/exclusion criteria, i.e., clearly excluded ocular diseases.

The pathophysiological mechanisms contributing to edema are numerous and multifactorial [113]. In diabetes, retinal edema (DME) contributes to tissue fibrosis. Recruited cells, firstly monocyte-derived macrophages, and later activated innate immune cells, potentially contribute to fibrosis formation [150]. The unanswered question remains whether inflammation-associated lymphangiogenesis in PDR eyes is related to promoting clearance of macular edema or regulation of immune cell trafficking or both. Since inflammation-related lymphangiogenesis could potentially either aide or worsen the DME and/or PDR progression, further DME/PDR-related studies are urgently needed. Furthermore, the functional capacity of these lymphatic-like vessels remains to be studied. In a neovascular RVO disease that primarily does not involve inflammation, the ischemia-induced formation of lymphatic-like vessels is also an interesting finding in need of further investigation which could open the avenue for novel therapeutic approaches towards other ischemia-driven eye diseases, such as neovascular sickle cell retinopathies.

Answering the numerous critical questions in the PDR field requires advances in translational research, including the investigation of the PDR tissue microenvironment communication. Pathological PDR neo(fibro)vascular tissues and patient-matched vitreous samples are invaluable material for this purpose. In addition to the retinal vasculature, the pathological DR mechanisms involve the ON, neural retina, RPE and choroidal vasculature [151]. Investigating the pathological mechanisms in these different compartments, will be of importance for understanding PDR pathophysiology. For example, the significance of factors secreted by the RPE deserves to be studied more in the future [152].

As PDR is a multifactorial and complex disease, multidisciplinary efforts and a holistic approach to the disease will be beneficial. Laborious scientific efforts are needed to understand the contribution of lymphatic-like formation, EPC involvement, immunity, inflammation and fibrosis together with the vitreal and tissue microenvironment in the PDR disease initiation, progression, and resolution. In the future, single cell transcriptomics will hopefully reveal novel aspects of this disease and the PDR neovessels of the human diabetic eye.

Finally, although neurodegeneration, inflammation and oxidative stress have been implicated in the pathogenesis, DR is still broadly treated as a purely ischemic vascular disease. Particularly, as anticipated by Yang et al., “lymphangiogenesis has now been implicated in many diseases for which treatments with angiogenic inhibitors have failed, and several research groups are on the brink of uncovering pathophysiological mechanisms involving lymphangiogenesis” [36]. Recently-emerged novel features underlying diabetes-induced injuries such as inflammation, lymphatic involvement and EPC function can have great impact on response/resistance to current therapies, as well as provide the basis for the development of new treatments to restore retinal homeostasis, or even prevent DR progression.

## Figures and Tables

**Table 1:** Positive and negative findings on the existence of lymphatic vessels in ocular structures.

		Positive Findings in Physiological Conditions		Negative Findings in Physiological Conditions		Positive Findings in Pathological Conditions	
		Marker	Reference	Marker	Reference	Marker	Reference
Conjunctiva	Vascular	Lyve1 Lyve1 * PDPN PDPN * VEGFR3	[52,64,65] [53,61,66] [52,64,65,67,68] [53,61] [64]			Lyve1 PDPN ^†^	[69] [70]
	Extra-vascular						
Cornea	Vascular			Lyve1 Lyve1 * PDPN VEGFR3 Prox1	[64] [66] [64] [64,71] [71]	Lyve1 PDPN VEGFR3	[64,72] [64,67,73] [64]
	Extra-vascular						
Lacrimal gland	Vascular					PDPN ^†^	[70]
	Extra-vascular						
Iris	Vascular	VEGFR3	[74]	VEGFR3 Prox1	[71] [71]	PDPN	[73]
	Extra-vascular	Lyve1 ^‡^ PDPN VEGFR3	[71], [74] ^‡^ [71,74] [74]	VEGFR3 Prox1	[71] [71]		
Trabecular meshwork	Vascular						
	Extra-vascular	Lyve1 Lyve1 * PDPN PDPN *	[71] [61] [71,75] [61]	VEGFR3 Prox1	[71] [71]	PDPN	[76]
Schlemm’s canal	Vascular	Prox1	[46]	Lyve1 * PDPN *	[61] [61]		
	Extra-vascular			PDPN *	[61]		
Ciliary body	Vascular	Lyve1 PDPN immunoEM	[52] [52] [52]	Lyve1 PDPN PDPN *	[74] [74] [61]	Lyve1 PDPN	[77] [73,77]
	Extra-vascular	Lyve1 PDPN VEGFR3	[71,74] [71,74] [74]	PDPN *	[61]		
Macula	Vascular						
	Extra-vascular						
Retina	Vascular			Lyve1	[57]		
	Extra-vascular	PDPN *	[61]	Lyve1	[57]		
Choroid	Vascular	Lyve1 PDPN ^§^ VEGFR3 *^,§^ VEGFR ^3,§^	[55] ^§^ [55] ^§^ [55] ^§^ [55] ^§^	Lyve1 PDPN PDPN * VEGFR3 Prox1	[56,57] [56] [61] [56] [56]		
	Extra-vascular	Lyve1 ^‡^ Lyve1 *^,§^ Lyve1 * PDPN PDPN *^,§^ VEGFR3 *^,§^ Prox1 *^,§^	[57] ^‡^ [55] ^§^ [61] [57] [55] ^§^ [55] ^§^ [55] ^§^	VEGFR3 Prox1 PDPN *	[56] [56] [61]		
Sclera	Vascular			Lyve1 Lyve1 * PDPN PDPN *	[54,57] [53] [54] [53]	PDPN	[73]
	Extra-vascular	Lyve1 ^‡^	[54] ^‡^, [57]	PDPN	[54]		
Optic nerve	Vascular	Histology Lyve1 PDPN	[60] [59] [59]	Lyve1	[62]	PDPN ^†^	[70]
	Extra-vascular	Lyve1 ^‡^ Lyve1 ^‡^ Lyve 1 * PDPN *	[57], [62] ^‡^ [62] ^‡^ [61] [61]			PDPN ^†^	[70]

* fetal, ^†^ finding documented with no reference to pathology, ^‡^ also positive for macrophage markers CD68 or Iba1, ^§^ highly contested finding.

**Table 2:** Clinical trials for retinal neovascular diseases, Clinicaltrials.gov, November 2018.

Mechanism	Drug	Indication	Mode of Administration	Phase	Clinical Trial Identifier
Inhibitor of sodium-hydrogen exchanger NHE3, anti-angiogenic	Squalamine Lactate	PDR	Eye drop	Phase II	NCT01769183
Soluble VEGFR1 (adenoviral)	rAAV.sFlt-1	wet AMD	Subretinal injection	Phase I	NCT01494805
β1-AR and β2-AR blocker	Propranolol	ROP	Eye drops	Phase II	NCT02504944
28-mer RNA aptamer against VEGFA-165	Pegaptanib	IN, DR	Intravitreal injection	Phase I	NCT00295828
Soluble VEGFR3	OPT-302	wet AMD	Intravitreal injection	Phase I	NCT02543229
Analog of cortisol acetate	Anecortave Acetate	AMD	sub-tenon injection	Phase II	NCT00211484
anti-VEGF/anti-angiopoietin-2 bispecific antibody	RO6867461/Faricimab	CNV/AMD	Intravitreal injection	Phase II	NCT02484690
Chimeric protein against Tissue Factor	hI-con1	CNV/AMD	Intravitreal injection	Phase II	NCT02358889
human monoclonal antibody against TNFα	Adalimumab	CNV/AMD	Intravitreal injection	Phase II	NCT01136252
human monoclonal antibody against IL-2R	Daclizumab	wet AMD	Intravenous	Phase II	NCT00304954
humanized monoclonal antibody against TNFα	Infliximab	wet AMD	Intravenous	Phase II	NCT00304954
mTOR inhibitor	Rapamycin	wet AMD	Oral	Phase II	NCT00304954
siRNA against VEGFR1	AGN211745	CNV/AMD	Intravitreal injection	Phase I/II	NCT00363714
Recombinant Human VEGF Receptor-Fc Fusion Protein	KH902	wet AMD	Intravitreal injection	Phase III	NCT01436864
siRNA against RTP801	PF-04523655	wet AMD	Intravitreal injection	Phase I	NCT00725686
anti-VEGF aptamer	EYE001	wet AMD	Intravitreal injection	Phase II/III	NCT00021736
tetracycline antibiotic	Doxycycline monohydrate	PDR	Oral	Phase II	NCT00511875
VEGFR inhibitor (inhibits also PDGFR, c-Kit and c-Fms)	PTK787	wet AMD	Oral	Phase I/II	NCT00138632
siRNA against CTGF	RXI-109	wet AMD	Intravitreal injection	Phase I/II	NCT02599064

PDR, proliferative diabetic retinopathy; AMD, age-related macular degeneration; ROP, retinopathy of prematurity; IN, iris neovascularization; CNV, choroidal neovascularization.

## Supplementary Materials

Supplementary Materials can be found at http://www.mdpi.com/1422-0067/19/12/4034/s1.

## Abbreviations

ACE	Angiotensin-converting Enzyme
BEC	Blood Endothelial Cell
bFGF	basic Fibroblast Growth Factor
CNV	Choroidal Neovascularization
CSF	Cerebrospinal Fluid
CTGF	Connective Tissue Growth Factor
CYR61	Cysteine-rich 61
DME	Diabetic Macular Edema
DR	Diabetic Retinopathy
EC	Endothelial Cell
ECM	Extracellular matrix
EPC	Endothelial Progenitor Cells
EPO	Erythropoietin
HA	Hyaluronic Acid
HMGB1	High Mobility Group Box-1
IL	Interleukin
LEC	Lymphatic Endothelial Cell
LECP	Lymphatic Endothelial Cell Precursor
LYVE1	Lymphatic Vessel Endothelial Hyaluronan Receptor-1
MCP-1	Monocyte Chemoattractant Protein-1
M-CSF	Macrophage-colony Stimulating Factor
MIF	Macrophage Migration Inhibitory Factor
M-LECP	Macrophage-derived lymphatic endothelial cell precursor
MMP	Matrix Metalloproteinase
NF-κB	Nuclear factor kappa-light-chain-enhancer of activated B cells
NLRP3	Nucleotide-binding oligomerization domain, leucine rich repeat and pyrin domain containing-3
NOD	Non-obese Diabetic
NPDR	Non-Proliferative Diabetic Retinopathy
OCT	Optical Coherence Tomography
OIR	Oxygen-induced Retinopathy
ON	Optic nerve
OPC	Oligodendrocyte Precursor Cell
OPN	Osteopontin
PDGF	Platelet-derived Growth Factor
PDPN	Podoplanin
PDR	Proliferative Diabetic Retinopathy
PEDF	Pigment Epithelium-Derived Factor
PKC	Protein Kinase C
PROX1	Prospero Homeobox Protein 1
PRP	Panretinal Photocoagulation
RPE	Retinal Pigment Epithelium
RVO	Retinal Vein Occlusion
SDF1	Stromal Cell-derived Factor-1
TGFβ	Transforming Growth Factor-β
TNFα	Tumor Necrosis Factor-α
TRD	Tractional Retinal Detachment
VEGF	Vascular Endothelial Growth Factor
VEGFR	Vascular Endothelial Growth Factor Receptor
VESC	Vascular Endothelial Stem Cell
VH	Vitreous Hemorrhage
