# Supplementary material for: Lymphatic Vascular Structures: A New Aspect in Proliferative Diabetic Retinopathy
Source: Int J Mol Sci. 2018 Dec 13;19(12):4034. doi: 10.3390/ijms19124034 (PMC6321212; doi:10.3390/ijms19124034)
Supplement: Supplementary file 1 [file ijms-19-04034-s001.zip › ijms-372046-supplementary/Supplementary materials (english edits)_EG.docx]

**Supplementary Video 1.** 3D volume reconstruction of a native FT stained by whole-mount immunofluorescence. Lyve1 (R&D Systems, Oxon, UK) (red) visualizes the lymphatic endothelium, Hoechst-33342 (Thermo Fisher Scientific, Cheshire, UK) counterstain (blue) visualizes the nuclei. The image was taken using an upright epifluorescence microscope with optical sectioning function and combined with a computer-controlled 1.3 megapixel monochrome CCD camera and image acquisition software, using a 20×, 0.8 NA, objective (Zeiss, Jena, Germany). The captured Z-stack was imported into Imaris software (Imaris, version 9.1.0, Bitplane, Zurich, Switzerland) and processed by volume and surface reconstruction of the Lyve1^+^ endothelium and of the nuclei. Video rendering was performed using same software.
